# Thyroid Tuberculosis Abscess: A Systematic Review of Diagnostic Pathways and Management Strategies

**DOI:** 10.3390/tropicalmed11030081

**Published:** 2026-03-15

**Authors:** Pranav Shivashankar, Praween Senanayake, Thomas Stephen Ledger, Nicholas Ngui

**Affiliations:** 1 Department of Otolaryngology, Head and Neck Surgery, Prince of Wales Hospital, Sydney 2031, Australia; 2 Department of Otolaryngology, Head and Neck Surgery, Westmead Hospital, Sydney 2145, Australia; 3 Department of Infectious Diseases, Westmead Hospital, Sydney 2145, Australia; 4 General Surgery, Blacktown and Mount Druitt Hospitals, Sydney 2148, Australia; 5 School of Medicine, The University of Sydney, Sydney 2050, Australia; 6 School of Medicine and Psychology, The Australian National University, Canberra 2601, Australia

**Keywords:** thyroid tuberculosis, thyroid tuberculosis abscess, thyroid abscess

## Abstract

Background: Thyroid tuberculosis abscesses (TTA) are rare manifestations of extrapulmonary tuberculosis, with the available literature consisting almost exclusively of individual case reports and small observational series. This systematic review aimed to evaluate current management strategies and associated clinical outcomes for TTA. Methods: Reports describing confirmed TTA, specified treatment regimens and clinical outcomes were systematically identified and synthesised from PubMed, Embase, Web of Science and Google Scholar from the period 1990 to 2025. Studies with suspected but unconfirmed cases were excluded. Risk of bias was assessed using the Joanna Briggs Institute tool. A total of 22 studies comprising 33 patients were included. Results: Significant diagnostic delays were seen. When diagnosis was established, standard four-drug anti-tubercular therapy (ATT) for at least 6 months emerged as the predominant first-line treatment. Surgical or percutaneous drainage procedures were typically reserved for large abscesses, treatment failure, acute suppurative presentations or suspected malignancy. Across published cases, lesion resolution and preservation of euthyroid function were reported in 92% of patients. However, the absence of comparative studies and the reliance on highly selected case material limit definitive conclusions and raise concerns about publication bias. Conclusions: TTA is a rare entity, with established treatment success with ATT, with surgery reserved for selected cases. Higher-quality comparative data are required to inform optimal management strategies.

## 1. Introduction

Thyroid abscess formation is an uncommon occurrence, accounting for approximately 0.1–0.7% of all thyroid pathology [1]. Tuberculosis (TB) remains one of the leading infectious causes of death worldwide, with an estimated 10.6 million new cases and 1.3 million deaths reported in 2022 [2,3]. Extrapulmonary tuberculosis (EPTB) accounts for approximately 15–20% of incident TB among immunocompetent adults and up to 50% among people living with HIV, with lymphatic, pleural, osteo-articular and genitourinary involvement most frequently described [3]. Although extrapulmonary tuberculosis constitutes a substantial proportion of the global TB burden, thyroid involvement remains exceedingly uncommon, accounting for well under 1% of reported extrapulmonary cases in most series [3]. Within this extrapulmonary spectrum, thyroid involvement is exceptionally rare. Reported cases of thyroid tuberculosis and thyroid tuberculosis abscess (TTA) represent only a minute fraction of documented TB infections, with most literature limited to isolated case reports and small series. The COVID-19 pandemic has further disrupted global TB control efforts, contributing to delayed diagnoses, treatment interruptions and increased mortality in several regions [2]. These pressures have heightened the importance of recognising atypical and extrapulmonary presentations of TB, particularly those that may mimic more common pathologies.

Although *staphylococcus* and *streptococcus* species are the most frequently implicated pathogens in thyroid abscesses, *mycobacterium tuberculosis* (TB) is a recognised, albeit rare, causative agent [3]. Thyroid tuberculosis is uncommon, and associated abscess formation represents an even rarer clinical entity that is frequently misdiagnosed [4]. Clinical presentations often mimic more common thyroid pathologies such as bacterial abscess or malignancy, leading to delayed diagnosis and potentially unnecessary surgery [4,5]. Imaging including ultrasound, CT and MRI have neither specific nor sensitive findings, with lesions often resembling nodules rather than abscesses, leading to delays in treatment and source control [5].

Current diagnostic modalities for tuberculosis include direct microscopy with Ziehl–Neelsen staining for acid-fast bacilli, mycobacterial culture on solid or liquid media, nucleic acid amplification tests (NAAT) such as Xpert MTB/RIF, and immunological assays including interferon-gamma release assays (IGRA) [4]. While smear microscopy provides rapid but non-specific evidence of mycobacterial infection, culture and molecular assays enable species-level identification and drug susceptibility testing. Immunological tests indicate prior exposure but cannot distinguish latent from active disease or confirm organ-specific involvement, underscoring the importance of microbiological confirmation in suspected thyroid tuberculosis [4].

Although Mycobacterium tuberculosis complex (MTBC) is presumed to be the causative organism in most published reports, the growing recognition of non-tuberculous mycobacteria (NTM) in soft-tissue, head and neck, and other extrapulmonary infections highlights the necessity of species-level microbiological confirmation in suspected thyroid tuberculosis [5].

The thyroid gland has several anatomical and physiological features that confer resistance to infection, including a fibrous capsule, rich vascular supply and the antibacterial properties of colloid within thyroid follicles, which together are thought to explain the rarity of clinically overt thyroid infection despite widespread TB endemicity [2,6]. Despite this, infections can occur, including with TB as the offending organism, usually in the context of immunocompromised patients and epidemiologic exposure [7]. In the absence of randomised trials or prospective cohorts, clinicians must rely on low-level evidence from case reports and small case series to guide decisions on management.

Consistent with the broader epidemiology of TB, most published thyroid tuberculosis abscess cases originate from high-burden countries such as India, Pakistan and China, with only sporadic reports from low-burden settings including the United Kingdom, United States, Spain and Ireland. This uneven geographic distribution likely reflects a combination of underlying TB incidence, access to advanced imaging and microbiology, and publication and language biases.

In our synthesis of 33 reported patients with thyroid tuberculosis abscess, no non-tuberculous mycobacteria were identified; all culture- or PCR-confirmed isolates were reported as MTBC, although incomplete microbiological work-up in some cases limits precise estimation of species distribution.

This systematic review synthesises published reports of thyroid tuberculosis abscesses using a thematic structure encompassing epidemiology, diagnostic pathways, medical therapy, surgical and drainage strategies and clinical outcomes. The purpose of the study is to compile and synthesise the available evidence to guide diagnosis and management of this rare clinical entity.

## 2. Materials and Methods

### 2.1. Search Strategies

**Figure 1:**
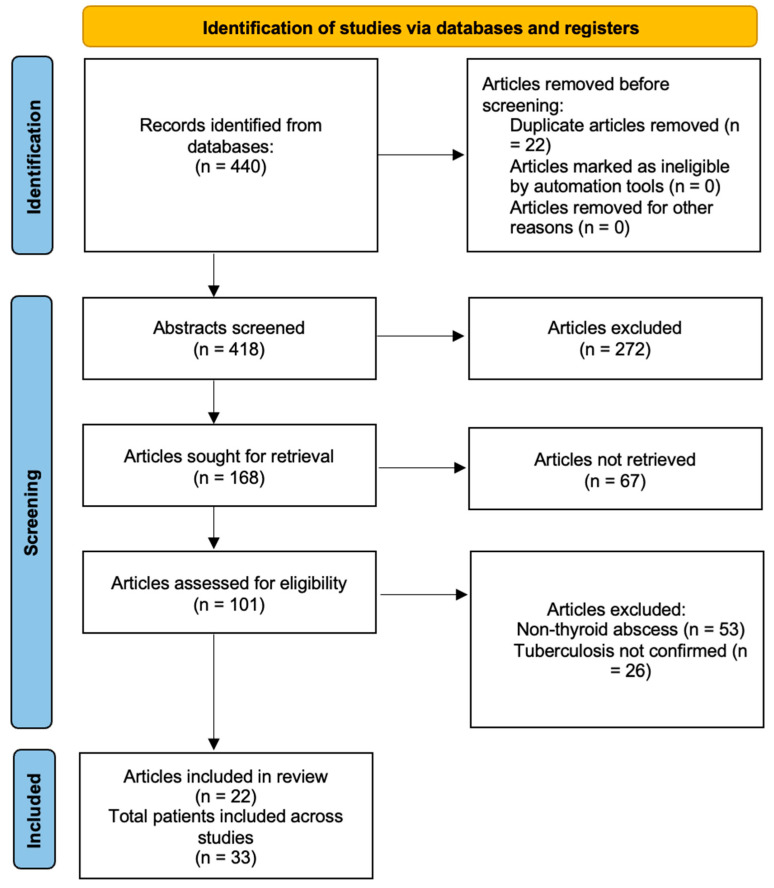
PRISMA 2020 Flowchart. Flowchart illustrating the identification, screening, eligibility assessment and inclusion of studies on thyroid tuberculosis abscess.

We conducted a comprehensive review of the literature in accordance with the Preferred Reporting Items for Systematic Reviews and Meta-Analyses (PRISMA) guidelines, identifying all relevant studies via PubMed, Embase, Web of Science and Google Scholar between January 1990 and December 2025 (Figure 1) [8]. The review was registered on the International Prospective Register of Systematic Reviews (CRD420251269263) and the search was performed on 15 December 2025. Search terms included ‘thyroid’, ‘thyroid abscess’, ‘thyroid tuberculosis’ and ‘thyroid tuberculosis abscess’, combined with database-specific subject headings.

The full PubMed search strategy was: (‘Thyroid Gland’[MeSH] OR thyroid OR goiter OR goitre) AND (‘Tuberculosis’[MeSH] OR tuberculosis OR tuberculous OR ‘Mycobacterium tuberculosis’ OR ‘extrapulmonary tuberculosis’ OR TB) AND (abscess* OR ‘cold abscess’ OR suppurative OR infection* OR collection OR thyroiditis). Equivalent strategies were adapted for other databases with the full search queries available in Supplementary Materials. The search strategy was limited to those published in English.

### 2.2. Study Selection and Data Extraction

Two investigators (PSh and PSe) independently performed searches and screened titles and abstracts. Titles and abstracts were screened for study design, population, presence of TTA, management strategies and clinical outcomes. A large language model assisted-tool, Elicit (Ought Inc., San Francisco, CA, USA; 2025), was used to support the initial data extraction from included studies. All extracted data were independently reviewed and verified by the authors against the original manuscripts to ensure accuracy. Final inclusion, data interpretation and synthesis were performed by the authors on individual full text review. Studies were limited to human research with available full-text articles, adult populations and articles reporting confirmed TTA, explicit management strategies and clinical outcomes.

Extracted data comprised of patient characteristics, diagnostic methods, treatment regimens, surgical and drainage procedures and outcomes. Outcomes of interest included clinical resolution, radiological resolution, recurrence, thyroid function at follow-up, complications and mortality. Other variables included demographics, immune status, diagnostic modality, abscess size and treatment characteristics. Extracted data were independently checked against the full texts by two reviewers, with disagreements resolved by discussion. Where consensus was not reached, data was discussed with the senior author (NN).

Included studies required microbiological, cytological or histopathological confirmation consistent with tuberculous infection. Where species-level confirmation was not available, cases were included if authors explicitly diagnosed thyroid tuberculosis based on accepted clinical and pathological criteria. Studies were excluded if thyroid involvement did not feature abscess formation, if no adult patients were reported, or if full text was unavailable in English.

Studies in which thyroid tuberculosis abscess was inferred solely from immunological tests (e.g., tuberculin skin test or interferon-gamma release assay) without microbiological, cytological or histopathological confirmation were excluded as insufficient to establish a definitive diagnosis.

### 2.3. Data Synthesis

Given the heterogeneity in diagnostic criteria, microbiological confirmation methods, definitions of treatment success, duration of therapy and follow-up reporting across included case reports and case series, quantitative pooling and formal meta-analysis were deemed methodologically inappropriate. The absence of comparator groups, inconsistent species-level confirmation and variability in outcome reporting would render pooled estimates potentially misleading and clinically non-interpretable. Accordingly, data were synthesised narratively and tabulated under the predefined thematic domains of epidemiology, patient characteristics, diagnostic pathways, microbiological confirmation, medical therapy, surgical and drainage strategies and clinical outcomes. Furthermore, the predominance of single-patient case reports introduces substantial reporting bias and selective outcome publication, further limiting the validity of any pooled quantitative estimate.

### 2.4. Critical Appraisal

Risk of bias assessment was performed for all included studies using the Joanna Briggs Institute (JBI) critical appraisal tools appropriate for case reports and case series [9].

## 3. Results

### 3.1. Overall Study Characteristics

Of the 168 full-text articles retrieved, 22 studies met inclusion criteria, comprising 33 patients. The majority were single-centre case reports, with only two case series including three or more patients; the largest series comprised nine patients. Publication years ranged from January 1990 to December 2025. All studies were observational and retrospective in design, and none included a comparator group or formal prospective follow-up. Most reported cases originated from high tuberculosis-burden countries—particularly India, Pakistan and China—with only sporadic reports from lower-burden settings such as the United Kingdom, United States, Spain and Ireland (Table 1).

Of the 272 excluded abstracts, the most common reasons were absence of thyroid involvement, absence of abscess formation, non-tuberculous pathology, insufficient diagnostic confirmation, duplicate reporting and non-English publication. Sixty-seven full texts were not retrievable despite institutional access attempts, reflecting the predominance of older case reports and regional publications. The majority of exclusions occurred at abstract screening due to lack of thyroid involvement or absence of abscess formation, consistent with the narrow focus of this review.

### 3.2. Epidemiology and Patient Characteristics

Across the pooled cohort, the median age at presentation was 46 years (range: 9–76 years old), with a slight female predominance of F:M = 1.2:1, where reported. Most patients were immunocompetent; however, 6 cases reported comorbidities including inflammatory myopathy on immunosuppressive therapy, COVID-19 related immunocompromise, HIV infection. Only one case had a history of drug susceptible pulmonary tuberculosis. No cases reported known tuberculosis exposure and no case reported a history of BCG vaccination.

### 3.3. Clinical Presentation and Disease Patterns

Patients with TTA most commonly presented with unilateral neck swelling (81% of reported cases), typically evolving over a period of 3–4 months, (range 7 days to 4 years) and was frequently associated with pain in 50% (Table 2). There was variability in systemic symptoms: fever occurred in 21% of patients, weight loss in 6% and night sweats in 17%, with these features more common among patients with coexistent pulmonary or disseminated TB. Local compressive symptoms were also common, including dysphagia in 4 patients, odynophagia in 1 patient and 1 patient with documented voice change, reflecting mass effect on adjacent aerodigestive structures. Despite this, there were no reports of dyspnoea or stridor, likely due to timely presentation and management.

On examination, a tender, ill-defined thyroid mass was reported in 33% of patients and cervical lymphadenopathy in 50% of patients, where it was assessed. Despite substantial gland involvement, baseline thyroid function was reported as euthyroid in 92%, with hypothyroidism and hyperthyroidism confined to small minorities of 0% and 8%, respectively. Collectively, this pattern of non-specific systemic symptoms and overlapping local signs meant that tuberculosis was rarely suspected at first presentation and many patients were initially investigated or treated for alternative diagnoses before confirmatory testing established thyroid tuberculosis abscess. Abscess size varied considerably, from 2 cm nodules to cold abscesses measuring up to 10 × 8 cm (Table 2).

### 3.4. Diagnostic Pathways and Misdiagnosis

Diagnostic evaluation typically commenced with basic laboratory studies and neck imaging once a thyroid mass or abscess was suspected, but these initial investigations seldom distinguished tuberculous from bacterial or neoplastic pathology. Inflammatory markers (e.g., ESR, CRP, white cell count) were recorded in 12 of the 22 studies, with elevated markers in all of patients in whom they were reported. Thyroid function tests were within the reference range in 23 of 25 patients (92%) for whom biochemical data were reported, underscoring that preserved biochemical function does not exclude significant infection.

Ultrasound served as the first-line imaging modality in 26 of 33 patients and commonly demonstrated a hypoechoic (13/26, 50%), heterogeneous (11/26, 42%) or mixed echogenic intrathyroidal collections. Additional sonographic features included calcifications (5/26, 19%), cystic change (4/26, 15%), irregular margins (4/26, 15%), internal vascularity (2/26, 8%) and anechoic areas (2/26, 8%). Cervical lymph node status was explicitly assessed in six studies, with lymphadenopathy present in three patients.

Cross-sectional imaging with CT or MRI was obtained in 25 of 33 patients. Among patients who underwent CT scanning, normal lung parenchyma was reported in 16 of 23 patients (70%), whilst cystic or hypodense thyroid lesions were described in 5 patients (22%) and extension to surrounding tissues in 6 patients (26%). MRI data demonstrated intermediate signal intensity in one patient and ring enhancing lesions in the other.

Diagnosis was established via fine-needle aspiration cytology (FNAC) in 17 of 33 (52%) of patients and following surgical biopsy in the remaining 16 (48%) of patients. Among FNAC samples with reported cytology, granulomatous inflammation and/or caseous necrosis was identified in 11 of 17 patients (65%), while non-specific inflammatory changes were described in 6 of 17 patients (35%), often necessitating repeat aspiration or subsequent histological confirmation. Ziehl-Neelsen staining for acid-fast bacilli was documented in 13 of 17 FNAC specimens (76%) and was positive in 10 of 13 patients (77%). Mycobacterial culture or molecular testing (e.g., PCR) was undertaken in 14 of 17 patients (82%) and yielded microbiological confirmation in all cases. When bacterial culture was positive, susceptibility was reported in 1 case, which was susceptible to rifampicin. In cases where FNAC was inconclusive or unavailable (16 of 33 patients, 48%), diagnosis was ultimately established on surgical histopathology following procedures such as hemithyroidectomy or lobectomy.

Across the pooled cohort, species-level confirmation of Mycobacterium tuberculosis complex (MTBC) by culture and/or MTBC-targeted molecular testing (such as PCR or Xpert) was documented in 11 of 33 patients (33%) (Table 3). The remaining cases were diagnosed on the basis of histopathological granulomas and/or acid-fast bacilli (AFB) staining from thyroid or nodal tissue without confirmatory species identification, or had incomplete microbiological reporting.

AFB microscopy was reported in 17 of 33 cases and was positive in 10 of 17 (59%), whereas mycobacterial culture was performed in 14 of 33 cases and yielded MTBC in 12 of 14 (86%). MTBC-targeted PCR (including Xpert) was undertaken in 6 of 33 patients and was positive in all cases (100%). Notably, one patient had culture-confirmed MTBC despite a negative smear result, highlighting the limited sensitivity of microscopy alone.

While granulomatous inflammation and AFB positivity are highly suggestive of mycobacterial infection, these findings are not species-specific and cannot reliably distinguish MTBC from non-tuberculous mycobacteria (NTM). The variability in diagnostic confirmation methods underscores substantial heterogeneity in microbiological evaluation across reports and has important implications for interpreting both the true burden of thyroid tuberculosis abscess and reported treatment outcomes. No confirmed cases of non-tuberculous mycobacteria were identified in the included cohort (Table 3).

### 3.5. Anti-Tubercular Drug Therapy

Anti-tubercular therapy (ATT) formed the primary treatment approach across the included reports, with standard four-drug therapy (isoniazid, rifampicin, pyrazinamide and ethambutol) used as first-line treatment in 31 of 33 patients for whom regimens were described. The intensive phase was most commonly administered for 2 months, followed by a continuation phase with isoniazid and rifampicin for a total duration of 6 months (71% of cases), although extended courses of 8–14 months were employed in cases of disseminated disease, slow radiological resolution or significant immunocompromise (14% for 8 months, 14% for 12 months). Data for adverse drug reactions and modification secondary to this was reported in 1 case only (4%), and no reports included mention of adherence to treatment. Overall, ATT, sometimes in conjunction with diagnostic aspiration, was associated with complete clinical and radiological resolution of the TTA in 27% of patients managed without more extensive surgery or drainage, reflecting its utility when diagnosis is established early and abscess size or airway compromise do not require immediate intervention. For the purposes of this analysis, ‘ATT alone’ refers to patients managed without thyroidectomy or formal surgical drainage, although some underwent diagnostic aspiration.

Surgical and percutaneous drainage procedures were reserved for selected patients, most often those with large or fluctuant abscesses, diagnostic uncertainty or failure to respond adequately to initial empiric antimicrobials. Across the cohort, 73% of patients underwent some form of intervention, ranging from needle aspiration in 15%, percutaneous or open incision and drainage in 19%, to hemithyroidectomy or total thyroidectomy in 62%, occasionally performed under the preoperative impression of malignancy, and with some patients receiving multiple interventions. Minimally invasive approaches (aspiration or catheter drainage) combined with concurrent ATT were usually sufficient to control infection, with complete resolution reported in 100% (*n* = 3 cases) of such cases and time to resolution of 2–6 months. Of the thyroidectomy/hemi-thyroidectomy cohort, 12.5% of patients had trialled alternative treatment first, whilst 87.5% went directly to surgery. This was dictated by high clinical suspicion for malignancy. Adverse events such as transient or permanent recurrent laryngeal nerve palsy or post-operative hypothyroidism were not reported.

### 3.6. Clinical Outcomes and Prognosis

Outcome data were variably reported, with complete clinical outcome data available for 15 patients, recurrence data for 11 patients, thyroid function outcomes for 25 patients and mortality data for 28 patients. Complete clinical resolution of local symptoms (pain, neck swelling and compressive features) was documented in 68% of patients overall, with radiological resolution of the thyroid abscess reported in 75% of cases over a median follow-up duration of 6 months. Time to symptomatic improvement was not recorded with any specific timeframes, however, complete radiological resolution typically required 6–12 months. Recurrence of thyroid abscess or need for re-intervention was rare, in only 18% (*n* = 2/11) of patients experiencing recurrence, and a reintervention rate of 12% (*n* = 3/25) during the reported follow-up period for the cohort in total.

With respect to thyroid function, the majority of patients remained euthyroid (*n* = 25, 92%) at last review, whereas hyperthyroidism developed in 8% (*n* = 2/25). Procedure-related complications were infrequently described; there were no reports of wound infection or other surgical morbidities. Mortality was rare, occurring in 1 patient (1/28, 3.6%) in the context of disseminated tuberculosis, severe immunosuppression, *Nocardia* spp. coinfection and multi-organ failure secondary to aspiration pneumonia [16].

### 3.7. Quality Assessment

Overall, study quality was low to moderate, reflecting the predominance of single patient case reports and small case series (Table 1). Common limitations included incomplete reporting of follow-up, inconsistent outcome definitions and limited detail regarding diagnostic confirmation and treatment protocols. Due to the nature of the included studies with predominance of case reports, formal statistical assessment of reporting bias and risk-of-bias tools for comparative studies were not applicable.

## 4. Discussion

This systematic review highlights TTA as a rare but clinically significant manifestation of extrapulmonary tuberculosis. Across published reports, TTA predominantly affected middle-aged patients (median age 46 years), with a slight female predominance, most commonly presenting as unilateral neck swelling with insidious onset and half of patients having detectable cervical lymphadenopathy. Systemic features classically associated with tuberculosis—such as weight loss, fever, and night sweats were often absent or non-specific, and the majority of patients were biochemically euthyroid at presentation. Whilst most patients had general epidemiologic exposure, specific tuberculosis exposure was infrequently mentioned and only one patient had a past history of pulmonary tuberculosis. This combination of indolent local symptoms, preserved thyroid function and non-specific systemic features likely explains the frequent misdiagnosis of TTA as thyroid malignancy or acute suppurative thyroiditis [4,10,25].

### 4.1. Diagnostic Challenges and Misdiagnosis

Diagnostic delay emerged as a central theme across the included studies. Radiological investigations were almost universally performed early in the diagnostic work up, but demonstrated limited sensitivity. Ultrasound findings including hypoechoic or heterogeneous nodules, irregular margins, cystic change and internal calcifications overlap substantially with malignant thyroid nodules and bacterial abscesses. Similarly, CT and MRI were useful for localising and delineating lesion extent and adjacent tissue involvement, however rarely provided features specific enough to indicate tuberculosis. Furthermore, when conducted, pulmonary imaging was frequently normal, highlighting that absence of pulmonary disease does not exclude thyroid tuberculosis and should not deter targeted microbiological investigation. None of the included reports provided MTB lineage or genotyping data; therefore lineage-specific associations with TTA cannot be assessed.

FNAC was the most valuable diagnostic modality when utilised, particular when paired with Ziehl-Neelsen staining, mycobacterial culture or molecular testing. For patients for whom FNAC was diagnostic for granulomas or acid-fast bacilli, it allowed for early initiation of ATT [4,12,15,19,28]. However, FNAC was performed in just over half of reported cases, and even when undertaken, yielded non-diagnostic results in a substantial minority, highlighting the general limitations of this modality, including operator skill, cystic component of the lesion and criteria used to judge the adequacy of the specimen [28]. These limitations often precipitated diagnostic thyroidectomy, especially in cases with suspicious imaging or inconclusive cytology. The high rate of surgical diagnosis observed appears to reflect diagnostic uncertainty rather than failure of medical management, underscoring the importance of early FNAC with mycobacterial testing when TB is a plausible differential.

### 4.2. Management Strategies

Once identified, management patterns across published cases consistently pursued ATT. Standard four-drug therapy for drug-susceptible TB administered for approximately six months was associated with high rates of clinical and radiological resolution—in line with current World Health Organisation Guidelines for extrapulmonary tuberculosis not involving the central nervous system, bones, or joints [29]. Current recommendations for first line therapy includes 6-month regimens with rifampicin, isoniazid, pyrazinamide and ethambutol, with shorter regimens such as a four-month rifapentine and moxifloxacin containing regimens only recommended for patients with pulmonary disease alone [29]. For drug resistant TB, recent updates recommend an all oral regimen containing bedaquiline, pretomanid, linezolid and moxifloxacin based regimens of six months duration, with careful monitoring of toxicity and adherence support, for patients with extrapulmonary TB that is not disseminated and is not in the context of an HIV infection and some additional considerations. As this review highlights, TTA is exceptionally uncommon, and we would advocate for discussion at a regional TB multidisciplinary meeting to assess applicability of this regimen in the event of a drug resistant TTA. In our analysis, data on adverse drug reactions, treatment adherence, and regimen modification were limited, precluding more granular analysis of optimal duration or tolerability.

**Figure 2:**
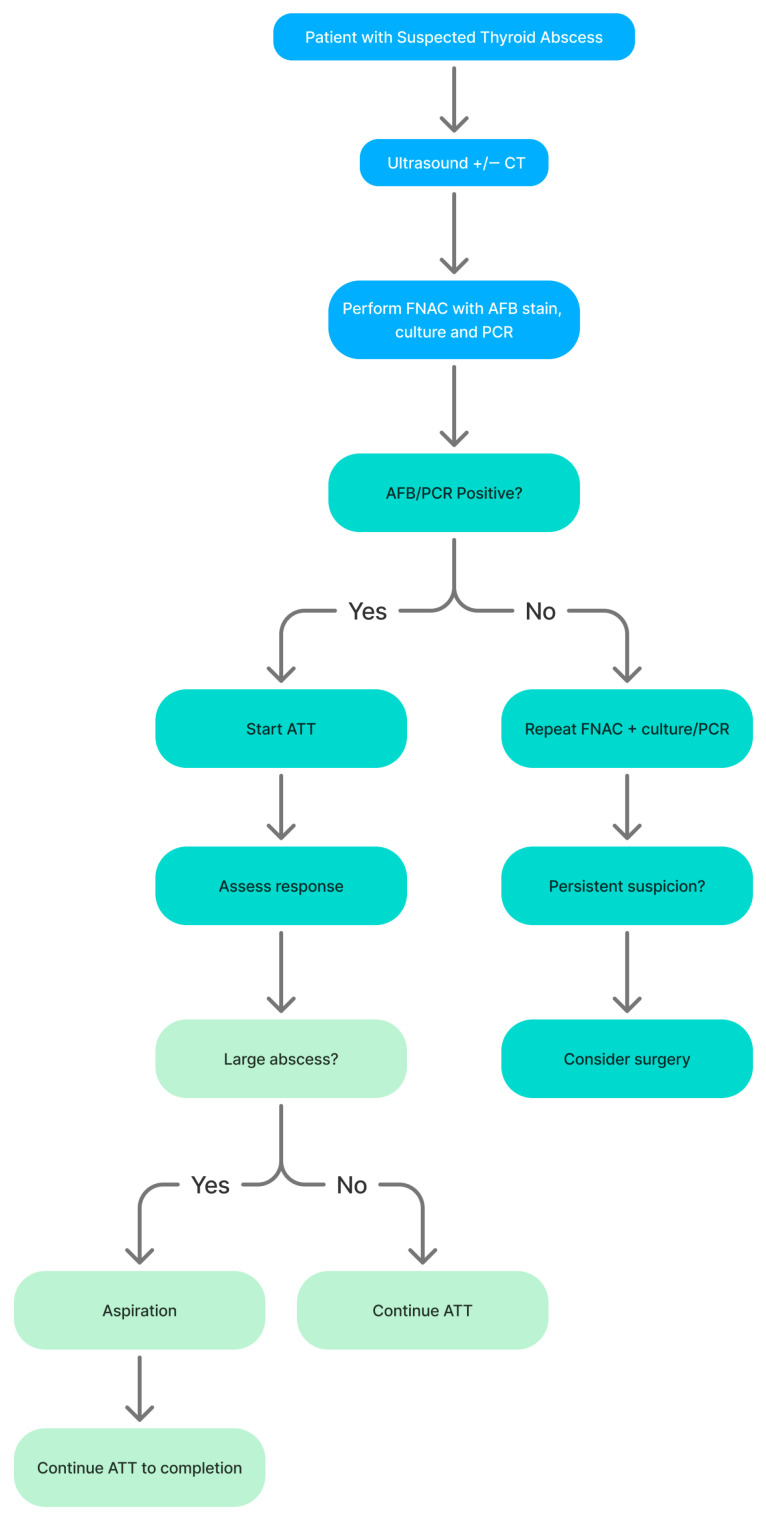
Schematic diagnostic pathway for suspected thyroid tuberculosis abscess, highlighting the central role of ultrasound-guided FNAC with AFB smear, culture and MTBC-targeted PCR, and the selective use of aspiration or surgery in addition to anti-tubercular therapy.

Adjunctive drainage procedures—either percutaneous aspiration or incision and drainage- were effective in selected patients with large or fluctuant abscesses and appeared sufficient when combined with ATT and it is something we would recommend be considered as a minimally invasive surgical approach. In contrast, thyroidectomy or hemithyroidectomy was frequently undertaken prior to confirmation of TB, often due to concern for malignancy or persistent diagnostic ambiguity. These findings therefore suggest that historical rates of thyroid resection likely overestimate the true need for surgery once the diagnosis of TTA is established. It follows that surgical intervention should therefore be reserved for clearly defined indications in these settings, including airway compromise, failure of ATT therapy, recurrent abscess formation or persistent suspicion of concurrent malignancy, or severe local paradoxical or immune reconstitution-type reactions. Based on the findings of this review, a proposed diagnostic and management algorithm for suspected thyroid tuberculosis abscess is illustrated in Figure 2.

### 4.3. Limitations and Future Directions

The conclusions of this review are constrained by the quality of the underlying evidence. All included studies were observational case reports of small case series, with the largest series comprising of only 9 patients and no comparative studies or randomised trials identified to evaluate relative outcomes of medical therapy alone versus combined medical and surgical approaches. This introduces substantial risk of selection and publication bias, and limits the ability to draw definitive conclusions regarding comparative effectiveness of medical versus surgical strategies. Heterogeneity in reporting further restricted synthesis and precluded quantitative analysis.

The predominance of single-patient case reports introduces reporting bias and limits generalisability. Furthermore, diagnostic heterogeneity and inconsistent species-level confirmation restrict the strength of epidemiological conclusions.

An important limitation of the available literature is the inconsistency of species-level microbiological confirmation. Although 11 of 33 patients (33%) in our cohort had MTBC confirmed by culture and/or MTBC-specific molecular testing, the remaining 22 cases (67%) were diagnosed on the basis of granulomatous histology and/or AFB positivity alone. These modalities confirm mycobacterial infection but are not species-specific and cannot reliably differentiate MTBC from non-tuberculous mycobacteria (NTM), which are increasingly recognised as causes of soft-tissue and head-and-neck infections. No included report identified NTM as the causative organism, and all culture- or PCR-confirmed isolates were reported as MTBC; however, the absence of comprehensive culture and molecular testing in one-third of patients means that misclassification of NTM as “tuberculous” thyroid abscess cannot be completely excluded.

Reporting of key variables was inconsistent, with minimal data on symptom duration, comorbidities, detailed imaging findings, cytological findings, drug regimens and follow-up intervals, which were incompletely documented in 62% of cases and several clinically important outcomes, including recurrence, long-term thyroid function and voice quality were either absent or, at most, qualitatively described. Procedure-related complications were rarely reported and amongst the 16 patients undergoing hemithyroidectomy or total thyroidectomy, there was no explicit documentation of transient or permanent recurrent laryngeal nerve injury. Given reported rates of recurrent laryngeal nerve injury of up to 6% and the debilitating effects of voice change, airway concerns and swallowing difficulty, these limitations are clinically significant [11,30,31]. These gaps, together with probable publication bias toward favourable or atypical presentations and under-reporting of treatment failures, mean that the high apparent success rates should be interpreted as optimistic estimates rather than robust measures of effectiveness.

Despite these limitations, several practical implications arise. Clinicians should maintain a high index of suspicion for TTA in patients presenting with unilateral thyroid swelling, particularly those from TB-endemic regions or with relevant risk factors. Early FNAC with routine requests for Ziehl–Neelsen staining, mycobacterial culture and molecular assays is essential to reduce diagnostic delay and avoid unnecessary thyroidectomy. Standard 6-month regimens for drug-susceptible TB remain appropriate based on current data, while extended courses of 8–14 months may be considered for disseminated disease or slow radiological resolution as reported in immunocompromised or disseminated cases [14,16,20,23]. Surgical resection and/or drainage should be reserved for patients with airway compromise, failure of conservative measures or suspicion of malignancy despite adequate cytology and microbiology, as the existing literature suggests that a substantial proportion of thyroidectomies were performed before TB was recognised.

No cases were identified from sub-Saharan Africa despite its high TB burden, likely reflecting underreporting, limited access to molecular diagnostics, and publication bias toward tertiary-centre case reports.

There is a strong need for more literature in this area, particularly case reports that more clearly report patient risk factors, exposure histories, and drug toxicity, and to understand the applicability of new oral regimens for drug resistant TB in TTA. Future research should prioritise prospective, multicentre data collection using standardised diagnostic and outcome measures, which may be possible at higher volume regional centres or via regional TB registries. This can help inform development of consensus diagnostic and management algorithms may assist clinicians in balancing timely initiation of medical therapy against the risks of delayed or missed malignancies, to help advance understanding and improve care for this rare but clinically important condition.

## 5. Conclusions

The existing literature supports standard ATT as the foundational treatment for TTA, with surgery reserved for large, suppurative or diagnostically uncertain lesions. A thematic synthesis highlights that early FNAC-based diagnosis, attention to immune status, disease extent and judicious use of drainage or resection together underpin favourable outcomes, despite the low quality of evidence. Future work should focus on systematic data collection and clearer protocols to minimise unnecessary thyroidectomy and optimise care in both endemic and non-endemic settings.

## Figures and Tables

**Table 1:** Summary of published case reports and case series describing thyroid tuberculosis, including study design, number of patients, geographic setting and classification as primary thyroid involvement or part of disseminated TB, defined as TB affecting more than one organ.

Study	Study Type	Number of Patients	Setting/Country	Primary Thyroid vs. Disseminated TB	Joanna Briggs Institute Risk of Bias Assessment
Reddy et al., 2016 [10]	Case report	1	India	Primary	Moderate
Magboo et al., 1990 [4]	Case report	1	USA	Primary	Low
Xiao et al., 2024 [5]	Case report	1	China	Primary	Low
Anwar et al., 2022 [6]	Case report	1	Germany	Primary	Low
Hussain et al., 2023 [7]	Case report	1	Ireland	Disseminated	Moderate
Majid et al., 2011 [11]	Case series	3	Pakistan	Primary	Moderate
Sun et al., 2022 [12]	Case series	9	China	Primary	Moderate
Sanehi et al., 2007 [13]	Case report	1	India	Primary	Moderate
Techopitayakul et al., 1998 [14]	Case report	1	Thailand	Primary	Moderate
Soni et al., 2015 [15]	Case report	1	India	Primary	Low
Rueda et al., 2024 [16]	Case report	1	Colombia	Disseminated	Moderate
Pachipala et al., 2024 [17]	Case series	2	India	Primary	Moderate
Bahgat et al., 2012 [18]	Case report	1	UK	Primary	Low
Kumar et al., 2013 [19]	Case report	1	UK	Disseminated	Low
Dv et al., 2017 [20]	Case report	1	India	Disseminated	Low
Zendah et al., 2015 [21]	Case report	1	Tunisia	Primary	Moderate
Azman et al., 2021 [22]	Case report	1	Malaysia	Primary	Low
Kandhasamy et al., 2016 [23]	Case report	1	India	Disseminated	Low
Srinivas et al., 2024 [24]	Case report	1	India	Disseminated	Low
Tong et al., 2025 [25]	Case report	1	China	Disseminated	Low
Modayil et al., 2010 [26]	Case report	1	UK	Primary	Low
Rojo-Abecia et al., 2021 [27]	Case report	1	Spain	Primary	Low

**Table 2:** Demographic characteristics, immunocompromising conditions, comorbidities, clinical presentation, and thyroid function of reported cases of thyroid tuberculosis presenting with thyroid abscess or mass. Data are summarized from published case reports and case series, with parameters including immune status, abscess size and presentation, and thyroid functional status at diagnosis.

Study	Age (Years)	Gender	Immunocompromising Conditions	Comorbidities	Abscess Presentation	Thyroid Function
Reddy et al., 2016 [10]	34	Male	Nil known HIV Negative	Nil reported	4 × 3 cm swelling Dysphagia	Euthyroid
Magboo et al., 1990 [4]	61	Female	Nil known	Prior Graves’ disease	3.0 × 2.8 × 1.6 cm mass	Euthyroid
Xiao et al., 2024 [5]	76	Male	Nil known	COVID 19	Painful neck swelling, fever	Euthyroid
Anwar et al., 2022 [6]	16	Female	Not reported	None reported	3 × 3 × 2 cm mass with skin ulceration	Notreported
Hussain et al., 2023 [7]	35	Male	Not reported	Not reported	2 cm neck abscess	Not reported
Majid et al., 2011 [11]	21, 51, 32	2 Female 1 Male	Not reported	Not reported	Thyroid nodules, fever, weight loss	Euthyroid
Sun et al., 2022 [12]	Median age 50 (range 43–64)	5 Female 4 Male	Not reported	Not reported	Neck masses, thyroid nodules	Euthyroid
Sanehi et al., 2007 [13]	45	Female	Not reported	Not reported	6 × 5 cm painless swelling	Euthyroid
Techopitayakul et al., 1998 [14]	38	Male	HIV	Nil other	2 × 3 cm neck mass, weight loss, fever	Not reported
Soni et al., 2015 [15]	38	Male	Not reported	Not reported	5 × 5 cm painless swelling	Euthyroid
Rueda et al., 2024 [16]	72	Female	Inflammatory myopathy	Hypertension, osteoarthritis	Painful right thyroid nodule	Low TSH, Normal FT4/T3
Pachipala et al., 2024 [17]	9 40	Both Male	Immunocompetent (9 year old) Anaemic (40 year old)	None	Neck swelling	Euthyroid
Bahgat et al., 2012 [18]	28	Female	Not reported	Not reported	Midline neck swelling with erythema, pain, fever	Euthyroid
Kumar et al., 2013 [19]	30	Male	Not reported	Pulmonary TB	Two cystic swellings	Euthyroid
Dv et al., 2017 [20]	46	Male	Nil known HIV negative	Disseminated TB (miliary)	10 × 8 cm cold abscess	Euthyroid
Zendah et al., 2015 [21]	47	Female	Not reported	Not reported	2 cm painful nodular swelling	Euthyroid
Azman et al., 2021 [22]	18	Female	Nil known	None	3.5 × 4.6 × 5.4 cm abscess	TSH 0.13 mU/L, FT4 21.1 pmol/L
Kandhasamy et al., 2016 [23]	45	Male	Nil known	Chronic HBV carrier, Hashimoto’s thyroiditis	8 × 9 × 8 cm left thyroid mass	Euthyroid
Srinivas et al., 2024 [24]	65	Female	Not reported	Family history of thyroid cancer Suspected previous pulmonary TB	6 × 5 cm swelling	Euthyroid
Tong et al., 2025 [25]	67	Female	Nil known	None	Two hypoechoic lesions (1.95 cm × 1.6 cm and 1.49 × 1.13 cm)	Euthyroid
Modayil et al., 2010 [26]	26	Female	Nil known	Polycystic ovaries	3.5 × 1.8 cm cystic mass	Euthyroid
Rojo-Abecia et al., 2021 [27]	50	Female	Nil known	None	3 cm thyroid mass	Not reported

**Table 3:** Diagnostic modalities and microbiological confirmation methods reported in included cases of thyroid tuberculosis abscess.

Study	Imaging	FNAC	Histopathology	AFB Stain	Culture	PCR	Species Confirmation	Basis of Diagnosis
Reddy et al., 2016 [10]	CT: pretracheal abscess mimicking thyroid swelling	Aspiration of pretracheal abscess	Not specified	Not specified	Not specified	Not specified	No	Clinical presentation as pretracheal abscess
Magboo et al., 1990 [4]	US: 3.0 × 2.8 × 1.6 cm hypoechoic mass with internal echoes	FNA: 3 mL milky white fluid	Surgery: granulomatous inflammation with multinucleated giant cells	Direct smear: few AFB positive (Kinyoun stain)	M. tuberculosis grew in culture	Not reported	Yes (M. tuberculosis culture positive)	FNA milky fluid with culture positive M. tuberculosis and surgical histology with granulomas and AFB positive
Xiao et al., 2024 [5]	PET-CT: FDG-avid right thyroid and nodes; US: anechoic lesion with punctate echoes; lung CT: miliary/patchy nodules	US-guided aspiration: mud-like fluid; smear inflammatory necrosis without malignant cells	No thyroid tissue histology (no surgical specimen)	Ziehl–Neelsen stain on aspirate positive for AFB	Mycobacterial culture not described	Xpert MTB PCR on thyroid aspirate positive	Yes (AFB smear positive and Xpert MTB-positive on thyroid aspirate)	Systemic TB with thyroid cold abscess; thyroid aspirate AFB+ and Xpert MTB+, clinical response to ATT
Anwar et al., 2022 [6]	US: multiple hypoechoic lesions; CT: hypodense cold abscess with gas	Needle biopsy: inflammatory cells	Lobectomy: granulomas with Langhans giant cells	Tissue AFB positive	Rapid culture test positive for MTB	Not specified (described as rapid culture)	Yes (confirmed M. tuberculosis)	Imaging with thyroid histology showing granulomas, AFB positive and culture positive for M. tuberculosis
Hussain et al., 2023 [7]	Imaging focused on chest/neck lump; thyroid involvement unclear	Not thyroid-specific	Neck tissue: granulomatous inflammation	Neck tissue ZN negative	BAL consistent with pulmonary TB	QuantiFERON positive	Unclear	Disseminated TB with cervical necrotic node/abscess (not definite intrathyroidal)
Majid et al., 2011 [11]	Case 1: thyroid scan cold nodule; Case 2: US left hypoechoic nodule; Case 3: thyroid nodule	Case 1: caseation necrosis; Case 2: follicular/Hurthle lesion; Case 3: TB cytology	Case 1: not done; Case 2: Hurthle adenoma with granulomas; Case 3: not specified	Case 1: pus AFB negative; Cases 2–3: not reported	Case 1: pus culture positive for TB bacilli; Cases 2–3: not reported	Not reported	Case 1: Yes (culture positive TB bacilli); Cases 2–3: No	Case 1: FNAC caseation with culture positive; Case 2: post-op histology; Case 3: FNAC with clinical response
Sun et al., 2022 [12]	Thyroid US in all; some nodules suspicious for malignancy; lung CT normal (no TB)	3/9 FNAC: atypical epithelial cells, malignancy not excluded; 6/9 no FNAC	All 9: post-op thyroid tissue with granulomatous inflammation and/or caseous necrosis	7/9 AFB stain positive; 2/9 negative	AFB culture recommended but not systematically reported for this series	PCR for M. tuberculosis genes positive in all 9	Yes (PCR-positive MTBC in all; histology compatible)	Post-op histology (granulomas/caseation) + AFB staining + universal PCR positivity for M. tuberculosis
Sanehi et al., 2007 [13]	X-ray/CT: peripherally enhancing low-density abscess right lobe	Yes: granuloma with caseous necrosis and Langhans cells	Not done (treated medically)	FNAC AFB positive (ZN stain)	FNAC aspirate culture positive for AFB	Not reported	Yes (AFB positive and culture positive)	FNAC granulomas with caseation, AFB positive, culture positive, and imaging
Techopitayakul et al., 1998 [14]	Thyroid scan: normal sized gland with midline trilobed cold nodule	Cystic content with inflammatory cells	Not reported	Thyroid pus AFB positive	Not explicitly reported	Not reported	Unclear	Clinical with imaging, thyroid pus AFB positive
Soni et al., 2015 [15]	US: heterogeneous hypoechoic lesion; CT: abscess with bulky strap muscles	Yes: pus aspirated	Not done (treated medically)	Pus AFB positive	Not reported (presumed not done)	Not reported	No	Clinical with imaging, FNAC pus AFB positive, and positive Mantoux
Rueda et al., 2024 [16]	US: right irregular nodule; CT: right lobe lesion extending to mediastinum	US-guided FNA: pus obtained	Thyroid biopsy: filamentous structures (Nocardia)	Thyroid pus AFB negative	FNA pus: Nocardia spp. and AFB compatible with MTB	Not specified beyond compatible with MTB	Unclear	Imaging with FNA pus showing Nocardia on histology/culture and AFB cultured as M. tuberculosis
Pachipala et al., 2024 [17]	Case 1: US/CT left lobe heterogeneous lesion with necrotic nodes; Case 2: US/CT multiloculated abscess left lobe	Both cases: FNAC with epithelioid granulomas and suppuration	Case 1: incision biopsy abscess; Case 2: necrotizing epithelioid granulomas	Not explicitly reported	Not specified if culture done	Both cases: TB-PCR on aspirate positive	Yes (TB-PCR positive)	Case 1: lymphadenopathy extending into thyroid with FNAC and TB-PCR; Case 2: disseminated TB with thyroid abscess and TB-PCR
Bahgat et al., 2012 [18]	CT: irregular cystic-like thyroid mass with enhancing walls	No FNAC (emergency drainage)	Abscess wall: epithelioid and Langhans cells, caseating necrosis	Not explicitly reported	Pus/abscess culture positive for M. tuberculosis (LJ)	Not reported	Yes (culture positive M. tuberculosis)	Imaging with acute abscess; histology with caseating granulomas and culture positive for M. tuberculosis
Kumar et al., 2013 [19]	US: two cystic swellings in right lobe; MRI: infective aetiology	US-guided FNAC: tubercular abscess	Not done (no surgery)	Not reported	Not reported	Not reported	No	Clinical (neck abscess with pulmonary TB) and FNAC cytology
Dv et al., 2017 [20]	US: large right-lobe nodule (8.6 × 5.9 × 8.3 cm) with cystic component	FNA/aspiration: used for microbiology	Not detailed	Aspirate AFB positive	Mycobacterial culture positive for MTB (MGIT)	Xpert PCR on aspirate: MTB positive, rifampicin-sensitive	Yes (Xpert MTB positive, culture MTB)	Disseminated TB with thyroid cold abscess: FNA AFB positive, Xpert MTB positive, culture MTB
Zendah et al., 2015 [21]	Neck US: cystic nodules in pyramidal lobe + bilateral hypoechoic cervical nodes; CXR normal	Not done (diagnosis via surgery)	Pyramidal lobe + node: necrotizing epithelioid granulomas with Langhans cells and caseous necrosis	Sputum AFB negative; tissue AFB rarely recognized and not demonstrated	No mycobacterial culture performed on specimen	Not done	No (histology-based; no stain positivity or culture)	Solitary thyroid mass + thyroid and nodal granulomas with caseous necrosis, no other TB focus, good response to ATT
Azman et al., 2021 [22]	US: multiloculated hypoechoic collections; CT: rim-enhancing abscess extending to prevertebral	FNA: frank pus aspirated	Surgery: xanthogranulomatous inflammation; no granulomas/Langhans	Pus AFB negative	Pus/tissue: S. anginosus, E. corrodens; no MTB isolated	TB PCR on pus positive for M. tuberculosis	Yes at DNA level (PCR positive) but no viable MTB culture	Imaging with thyroid abscess pus showing TB PCR positive in context of acute suppurative thyroiditis
Kandhasamy et al., 2016 [23]	US, CT: large heterogeneous left-lobe abscess with air loculi	Yes; necrosis, AFB negative	Core biopsy and surgery: caseous necrosis, granulomas with Hashimoto thyroiditis	FNAC AFB negative; core biopsy AFB positive	Aspirate culture MTB negative	Not done	No (AFB positive, MTB culture negative)	Clinical with imaging, core biopsy caseous necrosis AFB positive, and surgery histology
Srinivas et al., 2024 [24]	Not detailed beyond neck swelling	Yes: granulomas, atypical cells (Bethesda III)	Total thyroidectomy: nodular goiter with focal granulomas	Not reported	Not reported	Not reported	No (histology-based; pulmonary scarring post-op)	FNAC atypical cells with total thyroidectomy showing focal granulomas and post-op CT lung scarring
Tong et al., 2025 [25]	US: two hypoechoic lesions left lobe (C-TIRADS 4A)	US-guided FNA: suspected malignancy	Left thyroidectomy: granulomatous inflammation with caseous necrosis	Not reported	Not reported	Not reported	No (histology-based diagnosis)	Surgical specimen with granulomatous inflammation and caseous necrosis
Modayil et al., 2010 [26]	US: 35 × 18 mm cystic mass with internal echoes; abnormal level II nodes	US-guided FNA: 10 cc frank pus	No thyroid surgery	Not reported	Pus culture positive for M. tuberculosis	Not reported	Yes (culture positive M. tuberculosis)	Imaging with FNA pus and culture positive for M. tuberculosis
Rojo-Abecia et al., 2021 [27]	US: 3 cm heterogeneous nodule left lobe with irregular borders	Yes: follicular lesion (Bethesda III)	Hemithyroidectomy: granulomas with multinucleated giant cells	Surgical specimen ZN positive (Ziehl-Neelsen)	Not reported	Not reported	No (AFB positive on ZN stain of surgical specimen, not on culture or PCR)	Surgical specimen histology with granulomas and AFB positive (ZN)

## Data Availability

All data were obtained from preexisting published data available freely online.

## Supplementary Materials

The following supporting information can be downloaded at: https://www.mdpi.com/article/10.3390/tropicalmed11030081/s1, Table S1: Full database search strategies used for PubMed/MEDLINE, Embase (Ovid), Web of Science, and Google Scholar.

## Abbreviations

The following abbreviations are used in this manuscript:

TB	Tuberculosis
ATT	Anti-tubercular therapy
FNAC	Fine-needle aspiration cytology
CT	Computed Tomography
MRI	Magnetic Resonance Imaging
JBI	Joanna Briggs Institute
PRISMA	Preferred Reporting Items for Systematic Reviews and Meta-Analyses
