# Supplementary material for: Thyroid Tuberculosis Abscess: A Systematic Review of Diagnostic Pathways and Management Strategies
Source: Trop Med Infect Dis. 2026 Mar 15;11(3):81. doi: 10.3390/tropicalmed11030081 (PMC13030784; doi:10.3390/tropicalmed11030081)
Supplement: Supplementary file 1 [file tropicalmed-11-00081-s001.zip › tropicalmed-4131202-supplementary.pdf]

Supplementary Materials:

Table S1: Full database search strategies used for PubMed/MEDLINE, Embase (Ovid), Web of Science, and Google Scholar.

**a)**	**Pubmed/MEDLINE:**<br>("Thyroid Gland"[Mesh] OR thyroid OR goiter OR goitre)<br>AND<br>("Tuberculosis"[Mesh] OR tuberculosis OR tuberculous OR "Mycobacterium tuberculosis" OR "extrapulmonary tuberculosis" OR TB)<br>AND<br>(abscess* OR "cold abscess" OR suppurative OR infection* OR collection OR thyroiditis)
**b)**	**Embase (Ovid)**<br>(exp thyroid/ OR thyroid.mp. OR goiter.mp. OR goitre.mp.)<br>AND<br>(exp tuberculosis/ OR tuberculosis.mp. OR tuberculous.mp. OR "Mycobacterium tuberculosis".mp. OR extrapulmonary tuberculosis.mp. OR TB.mp.)<br>AND<br>(abscess*.mp. OR "cold abscess".mp. OR suppurative.mp. OR infection*.mp. OR collection.mp. OR thyroiditis.mp.)
**c)**	**Web of Science**<br>TS=(thyroid OR "thyroid gland" OR goiter OR goitre)<br>AND<br>TS=(tuberculosis OR tuberculous OR "Mycobacterium tuberculosis" OR "extrapulmonary tuberculosis" OR TB)<br>AND<br>TS=(abscess* OR "cold abscess" OR suppurative OR infection* OR collection OR thyroiditis)
**d)**	**Google Scholar**<br>thyroid AND tuberculosis AND abscess
